# Characterization of the Antibacterial Activity of an SiO_2_ Nanoparticular Coating to Prevent Bacterial Contamination in Blood Products

**DOI:** 10.3390/antibiotics11010107

**Published:** 2022-01-14

**Authors:** Sahra Fonseca, Marie-Pierre Cayer, K. M. Tanvir Ahmmed, Nima Khadem-Mohtaram, Steve J. Charette, Danny Brouard

**Affiliations:** 1 Héma-Québec, Medical Affairs and Innovation, 1070, Avenue des Sciences-de-la-Vie, Quebec, QC G1V 5C3, Canada; Sahra.Fonseca@hema-quebec.qc.ca (S.F.); Marie-Pierre.Cayer@hema-quebec.qc.ca (M.-P.C.); 2 Department of Biochemistry, Microbiology and Bioinformatics, Faculty of Science and Engineering, Laval University, Quebec, QC G1V 0A6, Canada; Steve.Charette@bcm.ulaval.ca; 3 TriPhyll Inc., Mississauga, ON L4G 4X1, Canada; ahmmed.kmtanvir@gmail.com (K.M.T.A.); nkhadem@gmail.com (N.K.-M.); 4 Department of Chemistry, Faculty of Science and Engineering, Laval University, Quebec, QC G1V 0A6, Canada

**Keywords:** nanoparticles, toxicity, blood, transfusion reaction, infection, polyvinyl chloride, polyurethanes, silicone

## Abstract

Technological innovations and quality control processes within blood supply organizations have significantly improved blood safety for both donors and recipients. Nevertheless, the risk of transfusion-transmitted infection remains non-negligible. Applying a nanoparticular, antibacterial coating at the surface of medical devices is a promising strategy to prevent the spread of infections. In this study, we characterized the antibacterial activity of an SiO_2_ nanoparticular coating (i.e., the “Medical Antibacterial and Antiadhesive Coating” [MAAC]) applied on relevant polymeric materials (PM) used in the biomedical field. Electron microscopy revealed a smoother surface for the MAAC-treated PM compared to the reference, suggesting antiadhesive properties. The antibacterial activity was tested against selected Gram-positive and Gram-negative bacteria in accordance with ISO 22196. Bacterial growth was significantly reduced for the MAAC-treated PVC, plasticized PVC, polyurethane and silicone (90–99.999%) in which antibacterial activity of ≥1 log reduction was reached for all bacterial strains tested. Cytotoxicity was evaluated following ISO 10993-5 guidelines and L929 cell viability was calculated at ≥90% in the presence of MAAC. This study demonstrates that the MAAC could prevent bacterial contamination as demonstrated by the ISO 22196 tests, while further work needs to be done to improve the coating processability and effectiveness of more complex matrices.

## 1. Introduction

Bacterial contamination remains a leading cause of transfusion-transmitted infections (TTI) [1]. Platelet concentrates (PC) are a more frequent source of transfusion-associated bacterial sepsis than red cell concentrates (RCC; 1 in 50,000 vs. 1 in 500,000 transfusions), mainly because the PC storage conditions (at room temperature under continuous agitation) promote bacterial proliferation. The rate of TTI associated with PC transfusions is ~1 in 2000–3300 transfusions, whereas that associated with RCC transfusions is ~1 in 38,500 transfusions [1]. Serious reactions occur in 1% of blood transfusions, with fatal consequences occurring in 1 in 200,000-500,000 transfusion [2]. Recent work has shown that plastic materials (PM) used to manufacture blood storage bags are conducive to the formation of biofilms, which complicates the monitoring of bacterial contamination [3]. The most common skin pathogen responsible for PC contaminations is *Staphylococcus epidermidis*, which has the ability to form biofilms that, if undetected, can lead to severe (and sometimes fatal) septic reactions [4,5,6,7]. TTI in RCC are mainly caused by biofilm-forming bacteria, such as *Serratia marcescens*, *Serratia liquefaciens* and *Staphylococcus* spp. [8,9,10,11]. However, *Yersinia enterocolitica* remains the most frequent contaminant found in RCC because of its psychrophilic nature [12].

To reduce the prevalence of TTI, blood banks have implemented safety measures over the last decades that include donor screening, disinfection techniques, removal of the first milliliters of whole blood donation, universal leukocyte reduction, and bacterial contamination tests before transfusion [11]. Nevertheless, only between 20–40% of contaminated blood products can be detected using systematic, automated microbial culture systems, with most false negatives caused by subthreshold levels of bacterial contamination or undetected inner-wall biofilms [12,13,14,15,16,17,18,19,20]. This limitation can be addressed using pathogen reduction technologies (PRT), which have been implemented by many blood banks to reduce the likelihood of bacterial, viral, and parasitic contamination in blood components and, more recently, whole blood. PRT is primarily used in high-income countries, given its high implementation/operational costs [12,21,22,23]. This technology depletes a wide range of potential contaminants through the use of UV illumination, either alone or in combination with photoactive compounds that cross-link nucleic acids [22,23,24,25,26]. Although effective against a wide range of known (and some emerging) pathogens, PRT does not remove all contaminants and thus cannot yet be substituted for existing detection tests [22,23,24,25,26].

Nanostructures have emerged as a promising tool to prevent viral and bacterial infections [27,28,29,30,31]. The incorporation of nanoparticles (NP) into synthetic polymers becomes appealing given the wide variety of NP compositions, shapes, and surface processing functionalities that confer useful end-product properties [31]. Work surfaces preventing non-specific adsorption of proteins and microbes are of particular interest for personal protective equipment and for the development of medical devices, textiles, food packaging and storage containers, water purification systems, and marine equipment [32,33,34,35]. However, the use of nanoparticles, and more particularly metallic nanoparticles such as silver and copper, for biomedical applications may present certain health risks due to recognized cytotoxicity and its adverse effects on the lungs that can lead to pulmonary diseases. [36,37,38,39].

The “Medical Antibacterial Antiadhesive Coating” (MAAC; TriPhyll, Ontario, Canada) is an anti-bacterial, hydrophobic nanoparticular coating that was primarily designed for medical device applications. The potential antimicrobial and anticoagulant properties of the MAAC could also be utilized by blood banks or collection kit manufacturers as an inner-wall coating for various blood storage bags. Specifically, the MAAC consists of functionalized silica (SiO_2_) NP that are incorporated into polymeric matrices to generate multifunctional capabilities. In fact, the SiO_2_ NP surface is treated with polysiloxane. In this study, the NP are covalently linked to a polyurethane matrix to prevent leaking. The polyurethane matrix is generated by the interaction of NCO groups of a commercial aliphatic polyisocyanate with the hydroxyl groups of a polyol. The incorporation of inorganic NP into the surface coating offers several advantages in addition to a demonstrated antibacterial activity, including improved thermal and mechanical properties (without compromising those of the treated material) [30,40,41]. The hydrophobic coating acts both as a repellent (i.e., it prevents bacterial adhesion) and an antimicrobial agent through the recognition of bacterial adhesion proteins [42]. Therefore, the incorporation of NP within polymeric matrixes arouses interest for its use in multifunctional coating systems combining organic- and inorganic-related properties [41]. However, mechanical friction can cause NP leaking, which may lead to NP agglomeration and (potentially) a loss of activity [40]. While this can be prevented by modifying the surface of SiO_2_ NP [41], doing so might impact the mechanical or bioactive properties of the polymer.

The present work aims to assess the antibacterial activity of MAAC when applied on the surface of plastic materials used to manufacture storage bags for labile blood products (LBP) and other biomedical devices. Potential safety risks were also evaluated using an in vitro cytotoxicity assay.

To the best of our knowledge, this study represents the first evaluation of the antibacterial performance of a synergistically acting coating in the presence of blood products using a standardized procedure, or a testing matrix that more closely reproduces the expected conditions of future applications.

## 2. Materials and Methods

### 2.1. Preparation of Labile Blood Products

This study was approved by the research ethics committee at Héma-Québec, the blood operator in Québec, Canada. Briefly, whole blood donations were obtained from healthy volunteers who signed an informed consent form. Reveos collection kits (Terumo BCT, Lakewood, CO, USA) were used to collect 450 mL (±10%) of blood; donations were stored for 16–24 h at room temperature before being processed using the Reveos 3C standard protocol, as previously described [41]. RCC units were transferred through an in-line leukoreduction filter into final RCC storage bags made of polyvinyl chloride (PVC)-diethylhexyl phthalate (DEHP) containing 100 mL of a saline-adenine-glucose-mannitol (Terumo BCT) additive solution. Units were then stored at 2–6 °C until use. Compatible ABO platelet interim units were pooled, and leukoreduction was performed using the Reveos pooling set (Terumo BCT, cat. 41910) to create the final PC, which can be stored for up to seven days at room temperature under continual agitation. PC storage bags were made of PVC with a citrate-based plasticizer (butyryl trihexyl citrate [BTHC]).

Each blood component was isolated by centrifugation. For RCC, a volume of 25 mL per unit was centrifuged for 6 min at 2500× *g* and 4 °C. Red blood cells were separated from the supernatant and washed twice in 50 mL of sterile saline (0.9% NaCl). Cells and their corresponding supernatants were then resuspended in a final 25 mL volume of sterile saline. For PC, cells were isolated following the same procedure, this time substituting the centrifugation parameters of 10 min at 1430× *g* and 22 °C and using the resuspension solution of 25 mL of sterile 1X phosphate-buffered saline [PBS, Fisher].

### 2.2. Medical Antibacterial Antiadhesive Coating

The nanocomposite, polyurethane-based MAAC was provided by TriPhyll, Inc. Hydrophobic SiO_2_ nanoparticles, which are functionalized with polysiloxane, were dispersed in a two-part solvent-borne polyurethane coating. The liquid-phase MAAC was applied to the inner faces of PVC-DEHP containers (for RCC; Leukotrap RC system, Haemonetics, Boston, MA, USA) and BTHC-PVC (for PC; Reveos pooling set, Terumo BCT, Lakewood, CO, USA). The inner walls of the PC storage bags have one smooth and one textured surface. The MAAC was applied on the smoother side of the PC storage bags. For RCC units, both inner walls were textured, and the coating was applied on either side. For the other polymeric materials (PM) commonly used in biomedical devices (i.e., silicone and polyurethane), MAAC was applied directly on sections of 2500 mm [2]. Before each application, a 3-aminopropyl trimethoxy silane (APTMS) primer (Sigma; 2% APTMS, 6% distilled water and 92% isopropanol) was deposited on the surfaces to promote coating adhesion [42]. The liquid-phase MAAC was then added using a metallic rod specifically designed to apply a 50 µm-thick coating (with RD specialties) at the surface of each PM under study. Each MAAC-treated sample was then dried at 80 °C for one hour or until complete MAAC polymerization was reached.

### 2.3. Characterization Techniques

The MAAC was characterized using scanning electron microscopy (SEM; JEOL 6360LV). SEM samples were mounted on conductive supports and metallized with gold using sputter coating. In parallel, a 5 nm-thick gold and palladium coating was deposited on similar samples to assess the composition of the molecular surface using energy-dispersive X-ray spectroscopy (EDS). The size dispersion of SiO_2_ NP was evaluated using transmission electron microscopy (TEM; JEOL, JEM-1230). Samples were prepared by depositing a 5 μL suspension of the SiO_2_ NP and 5 μL of a 3% uranyl acetate solution on a copper grid previously coated with a carbon film. Residual liquid was absorbed using filter paper, and the grid was air-dried in a dust-free environment. The hydrodynamic diameter of SiO_2_ NP was determined using dynamic light scattering (DLS) measurements. The DLS results were analyzed using the Zetasizer system (Malvern). Experimental samples were obtained by diluting 10 μL of the SiO_2_ NP suspension in 1 mL of acetone. Each sample was measured in duplicate, and data were analyzed to obtain the hydrodynamic radius of the NP [43].

### 2.4. Antibacterial Activity

#### 2.4.1. Decontamination Sample

Sections of MAAC-treated PM, MAAC-untreated PM, and cover films were decontaminated by rubbing with ethanol. The residual contaminants were removed by passage through ethanol, then the excess solvent was removed by two passages through sterile 1X PBS. Before each test, the sections were dried using sterile gas and placed in a Petri culture dish as used in ISO 22196.

#### 2.4.2. Microorganisms and Growth Conditions

*Staphylococcus aureus* (ATCC 6538), *Escherichia coli* (ATCC 8739), *S. epidermidis* (ATCC 43862), *S marcescens* (ATCC 35984), *Klebsiella pneumoniae* (ATCC 13883), and *Enterococcus faecalis* (ATCC 47077) were obtained from the American Type Culture Collection. *S. aureus*, and *S. epidermidis* were subcultured in tryptic soy broth (BD Biosciences); a nutrient broth (NB; Difco) was used for *E. coli*, *S. marcescens*, and *K pneumoniae*; and a brain infusion broth (Fisher Scientific) was used for *E. faecalis*. All subcultures were incubated at 37 °C for 24 h. Lastly, bacterial cultures were frozen in 20% glycerol at −80 °C until use. When needed, each culture was subcultured twice onto nutrient agar and incubated at 37 °C for 24 h. Cultures were then resuspended in diluted NB or in LBP, as described in Table 1. Antibacterial activity against the bacteria of interest was evaluated in NB, PC or RCC, as described in Table 1.

#### 2.4.3. Antibacterial Activity on Polymeric Material Surfaces

Antibacterial activity was evaluated against bacteria loaded at 5.8 Log CFU/mL, following the ISO 22196, 2011 guidelines; the material and incubation temperatures tested for each contaminant are described in Table 1. The final bacterial concentration was adjusted based on the optical density at λ = 600 nm measured using spectrophotometry (Thermo Scientific, Genesys 10S UV-Vis). MAAC-treated and MACC-untreated sections of 2500 mm [2] were placed in direct contact with the bacterial inoculum and were then covered with 0.05 mm-thick polyethylene films (Delta Scientific). All samples were then subjected to a 24 h incubation period in a ≥90% relative humidity environment at the optimal growth temperature of the corresponding species (Table 1). Bacteria were recovered in a soybean casein digest broth with lecithin and polyoxyethylene sorbitan monooleate [44]. Viable bacteria were counted by plating different dilutions on plate count agar, incubating the plates for 24 h at 37 °C, and then visually counting the colonies. Consistent with the ISO guidelines, antibacterial activity was estimated using the following equation:

(1) $$\mathrm{Antibacterial}\;\mathrm{rate}\;(\%)=\frac{\left(N_{U}-N_{C}\right)\;}{N_{U}}\;\times \;100$$

where *N_U_* is the bacterial count obtained for the control sections after incubation, and *N_C_* is the bacterial count observed for the test conditions for the MAAC sections after incubation.

### 2.5. In Vitro Cytotoxicity

The MAAC cytotoxicity was evaluated using the mouse fibroblast cell line L-929 (ATCC CCL-1). Cells were placed in direct contact with the coating, in accordance with ISO 10993-5, 2009 guidelines. Cells were grown in the Roswell Park Memorial Institute medium (Thermo Fisher) supplemented with 10% fetal bovine serum (Sigma), without phenol red, at 37 °C/5% CO_2_. After a minimum of two passages, 1 × 10^5^ cells per well were deposited on MACC-treated and MACC-untreated coverslips in a 12-well plate. The plate was incubated for 24 h at 37 °C/5% CO_2_. A 1 mg/mL solution of 3-(4,5-dimethylthiazol-2-yl)-2,5-diphenyltetrazolium bromide (MTT; Molecular Probes) was added to each well for 2 h at 37 °C/5% CO_2_. Finally, the MTT solution was removed, and 100 μL aliquot of isopropanol (Fisher) was added to each well. After a 5 min agitation period, the optical density at λ = 570 nm was measured using a plate reader (BioTek, Synergy H1 microplate reader).

In parallel, viability was assessed using a double-staining fluorescent microscopy method (NucleoCounter^®^NC-250TM, ChemoMetec, La Jolla, CA, USA) based on acridine orange (AO) and 4,6-diamidino-2-phenylindole (DAPI; solution 18, ChemoMetec) following the manufacturer’s instructions. Viability was also assessed using a flow cytometry assay (Accuri C6 cytometer; BD, C6 Flow Cytometer) based on 7-Aminoactinomycin D (7-AAD, BD Bioscience); viability and cell concentration were determined following the manufacturer’s recommendations.

### 2.6. Statistical Analysis

Bacterial counts were converted to logarithmic values, which were analyzed using standard summary statistics (i.e., means and standard deviations [SD]). Acceptability criteria for the antibacterial activity and cytotoxic tests were obtained following ISO standards [45]. The antibacterial and cytotoxicity assays were performed three times for consistency. Microscopy data were analyzed using ImageJ [46]. Statistical analyses for bacterial growth inhibition were performed using XLSTAT 2021.2.1.1118. A Student two-sample t-test for means differences (α = 0.05) was used to identify statistical differences between the reference and the MAAC condition for each bacteria strain (*n* = 3/bacteria strain).

## 3. Results and Discussion

### 3.1. Characterization

**Figure 1:**
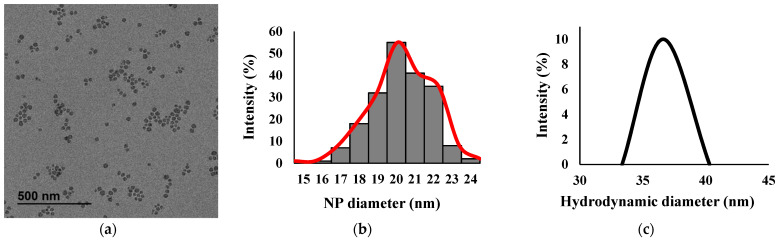
Characterization of functionalized SiO_2_ NP using TEM and DLS. (**a**) A representative TEM image, (**b**) size distribution calculated from TEM images of SiO_2_ NP, and (**c**) the DLS spectrum.

The functionalized SiO_2_ NP had a mean ± SD diameter of 20 ± 6 nm based on TEM image processing and a hydrodynamic diameter of 37 ± 3 nm based on DLS measurements (Figure 1 and Table S1). The 30% relative SD might be due to silane monomers reacting together during synthesis [47,48]. The observed size difference between the TEM images and the DLS measurements can generally be explained by differences in the sample preparation and analysis methods used in both techniques: TEM provides information on the NPs’ solid diameter, whereas the DLS data correspond to an interpreted hydrodynamic diameter that can be influenced by the NPs’ size and behavior in the suspension medium, which can in turn be affected by functionalized surface molecules [49,50].

**Figure 2:**
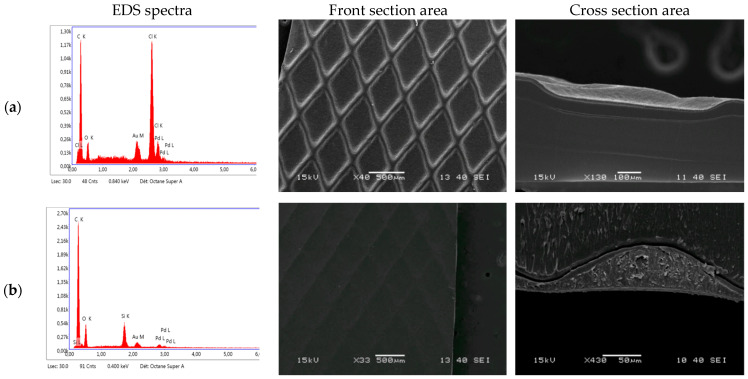
The SEM/EDS characterization of the RCC bag sections. The SEM images of the (**a**) uncoated and (**b**) MAAC-coated PVC-DEHP samples.

Once added to the MAAC polyurethane matrix, SiO_2_ NP appeared to aggregate during the drying phase (Figure 2b, white areas). The MAAC-treated storage bags presented a smoother and a more uniform surface than the MAAC-untreated bags (Figure 2 and Figure S1). The mean ± SD thickness of the dried MAAC was 9 ± 3 µm on the thinner sections of the textured PVC-DEHP bags and 52 ± 13 µm on the deeper sections (Figure 2 and Table S2). Similar results were obtained for the other PM considered (Figure S1).

Since the solvent accounts for 50% of the liquid-phase MAAC, the expected thickness of the coating after the drying step should be up to half that of the rod used for its applications owing to solvent evaporation. Because the rod only controls MAAC thickness in the thinner sections of the textured PVC-DEHP, the thickness may be greater at the deeper textured sections of the studied material. In addition, the relative amount of oxygen at the surface of the PVC-DEHP increased from 8 ± 2% to 12 ± 3% following the MAAC application, and the relative amount of silica increased from 0% to 2 ± 1% (Figure 2, Table S3). The 2 ± 1% relative intensity of silica for the MAAC-treated PVC-DEHP is not proportional to the total amount of SiO_2_ in the coating, which might be explained by the higher carbon chain concentration from the polyurethane matrix masking the silica signal. The polarizing electric field is unlikely to go through the entire MAAC layer [51]. Figure 2 also shows that there is a complete loss of the chlorine signal (from 8 ± 1% to 0%) following the MAAC application. The relative measure of carbon intensity did not change significantly between the reference and the MAAC conditions (85 ± 2% vs. 87 ± 3%, Figure 2 and Table S3). The strong increase in oxygen amounts and in the SiO_2_-related bands in the test conditions was used to confirm the presence of MAAC at the surface of the PM, as the coating chemical composition is mostly polyurethane, and carbon chains functionalized SiO_2_ nanoparticles in suspension. The presence of chlorine in the reference PVC-DEHP can be attributed to the chemical composition of PVC [52]. Indeed, the DEHP application may not be uniform at the surface of the reference section since the plasticizer is not covalently bound to the plastic material and is known to be partially released in RCC during storage [53]. The absence of chlorine-related bands in the MAAC-treated samples confirms the presence of a thin coating layer at the top of the PM. Carbon-related signals are expected to be predominant, as carbon is one of the major constituents of DEHP [54].

### 3.2. Antibacterial Activity

**Figure 3:**
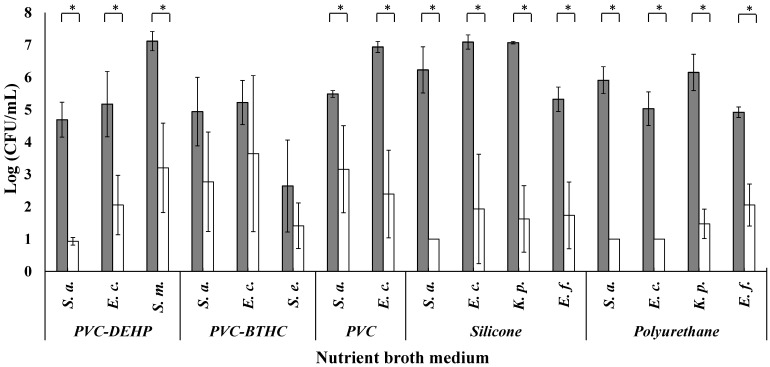
Logarithmic bacterial growth (mean ± standard deviation) in nutrient broth (1/500) with an inoculum of 6 × 10^5^ CFU/mL after 24 h in contact with untreated (reference) and treated polymeric materials (MAAC) (*n* = 3). *S. a.* = *Staphylococcus aureus*; *E. c.* = *Escherichia coli*; *S. m.*= *Serratia marcescens*; *S. e.* = *Staphylococcus epidermidis*; *K. p.* = *Klebsiella pneumoniae*; *E. f.* = *Enterococcus faecalis*. * Significant growth difference between the reference and the MAAC condition (Student *t*-test, α = 0.05, * *p*-value < 0.05).

The antibacterial activity of the MAAC was investigated using ISO 22196 given its potential application on the PM sections (including LBP storage bags) to prevent bacterial contamination and biofilm formation. This protocol is considered the gold standard when it comes to testing plastics and other non-porous surfaces with antimicrobial claims [50]. The antibacterial activity of the MAAC-treated PM against the four strains after a 24 h exposition period in nutrient broth (NB) is presented in Figure 3 and Figure S2 and Table 2. MAAC-treated silicone sections exhibited the highest antibacterial effect, with a log reduction between 3.6 ± 0.9 for *E. faecalis* (ref = 5.3 ± 0.4 log _CFU/mL_, MAAC = 1.6 ± 0.7 log _CFU/mL_; *p* = 0.001) and 5.5 ± 1.1 for *K. pneumoniae* (ref = 7.1 ± 1.6 log _CFU/mL_, MAAC = 0.0 ± 1.0 log _CFU/mL_; *p* = 0.001) (Figure 3). This high antibacterial activity is partially related to the significant bacterial growth observed in untreated silicone sections (Equation (1)). The resistance of silicone to various perturbations (e.g., oxidation, environmental degradation, heat, moisture, and various chemical assaults) makes it an optimal material for use in the biomedical industry. However, silicone is conducive to bacterial adhesion on its surface [55]. Reductions of similar magnitude were observed in the MAAC-treated polyurethane sections with a log reduction between 2.9 ± 0.8 for *E. faecalis* (ref = 4.9 ± 0.2 log _CFU/mL_, MAAC = 2.1 ± 0.7 log _CFU/mL_; *p* = 0.002) and 4.9 ± 0.4 for *S. aureus* (ref = 5.9 ± 1.0 log _CFU/mL_, MAAC = 0.4 ± 0.0 log _CFU/mL_; *p* < 0.0001). The strong antibacterial activity of the MAAC on the polyurethane sections could in part be attributable to the compatible chemical composition of the MAAC and polyurethane, providing better adhesion properties and stability over time.

For non-plasticized and plasticized PVC sections, the lowest log reductions were observed for both Gram-positive and Gram-negative bacteria going from 1 ± 1 log for *S.* *epidermidis* (ref = 2.6 ± 1.4 log _CFU/mL,_ MAAC = 1.4 ± 0.7 log _CFU/mL_) onto PVC-BTHC to 4.6 ± 1.5 for *E. coli* (ref = 6.9 ± 0.2 log _CFU/mL_, MAAC = 2.4 ± 1.4 log _CFU/mL_; *p* = 0.004) onto non-plasticized PVC (Figure 3 and Figure S2). The higher variability in the measured log reductions could be related to the instability of the PVC sections at higher temperatures: the release of gaseous HCl during polymer drying at 90 °C might have partially degraded the PM substrate [56]. Under control conditions, both PVC-DEHP and (in particular) PVC-BTHC were less favorable to bacterial growth than other PM, which may have contributed to the higher variability in antibacterial activity (Figure 3).

The lower antibacterial activity observed in the PVC-BTHC sections may be related to the non-adhesive nature of the plasticizer which may cause moderate bacterial growth under control conditions [9,57]. A number of studies have discussed the importance of the PM topography in respect to bacterial adhesion, and they have shown that textured surfaces may be beneficial for adhesion, although this cannot be generalized to all materials [58]. Indeed, bacterial adhesion is greatly influenced by the size and shape of bacteria [59,60]. The majority of platelet contaminants, such as *S. epidermidis*, may adhere to and grow better on textured rather than smooth surfaces (similar to those of PC storage bags) [58]. Typically, storage bags have at least one textured surface to prevent fusion during the sealing and sterilization processes [9]. However, in the current study, tests were only performed on the smoother side of platelet storage bags. In addition, it is less challenging to apply a uniform coating on smooth surfaces, which may lead to more reproducible results and less coating delamination. Bacterial growth tests were carried out on smooth and textured PVC surfaces, and the same low growth patterns were observed. The antibacterial activity calculation recommended by the ISO guidelines (Equation (1)) [50] involves the growth of the bacteria under control conditions. This comparison to the control makes it possible to normalize the data using the expected bacterial growth on each of the polymeric materials studied along with the external conditions of the experiment. The lower bacterial growth observed on the PVC-BTHC control sections could explain its weaker MAAC antibacterial activity [59]. In fact, one must consider that bacterial growth can also be affected by nutrient concentration, growth phase (i.e., either stationary or exponential), incubation time, and the starting inoculum concentration. These parameters are the four critical factors identified by Wigand et al. that can influence the outcome of antibacterial testing in accordance with the ISO-22196, 2011 guidelines [61].

Overall, the mean antibacterial activity was higher for the Gram-positive bacterium *S. aureus* on all PM (4.0 ± 1.4 log _CFU/mL_) than for the Gram-negative bacterium *E. coli* (3.7 ± 1.8 log _CFU/mL_) (Figure S2). This observation could be due to the hydroxyl groups at the surface of SiO_2_ NP interacting more extensively with the peptidoglycan saccharides at the surface of the Gram-positive bacteria [60]. This interaction could destabilize the bacterial membrane and induce cell death by osmotic shock [60]. The lower levels and reduced accessibility of peptidoglycan on the surface of Gram-negative bacteria could explain the observed difference in the results [59,60].

The MAAC antibacterial properties have been demonstrated in nutrient broth, with a >90% reduction of bacterial growth for all species and the PM of interest (coated polyurethane and silicone [99–99.999%] vs. coated plasticized PVC- [90–99.9%]) (Table 2 and Figure S2). The lower reduction factors observed for the plasticized PVC sections suggest that BTHC and DEHP may interact with the MAAC and partially prevent its antibacterial activity.

Taken together, these results show that the MAAC could be used to prevent bacterial contamination at the surface of biomedical materials. In addition, the MAAC might interfere with the metabolism of bacteria by subjecting them to a stressful environment. Indeed, bacteria colonies observed under test conditions were smaller than those observed under control conditions for all PM (Figure S3) [9].

**Figure 4:**
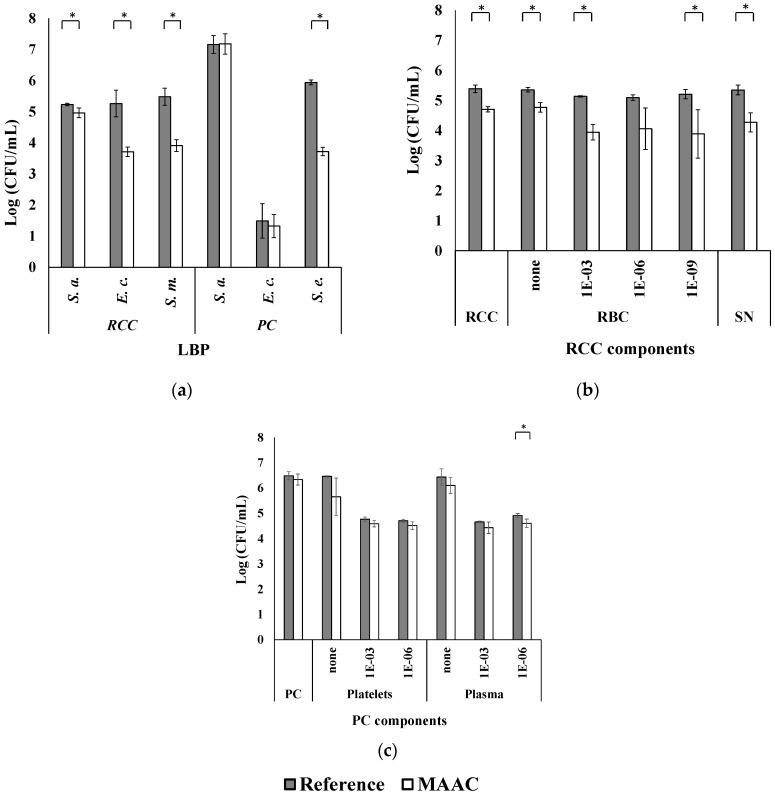
Logarithmic bacterial growth (mean ± standard variation) in blood products with an inoculum of 6 × 10^5^ CFU/mL after a 24 h contact period with untreated (reference) and treated polymeric materials (MAAC). (**a**) Logarithmic growth in RCC (PVC-DEHP) or PC (PVC-BTHC) matrices using three bacteria. Logarithmic bacterial growth of *S. aureus* in (**b**) RCC and (**c**) PC component dilutions in saline (*n* = 3). *S. a.* = *Staphylococcus aureus*; *E. c.* = *Escherichia coli*; *S. m.* = *Serratia marcescens*; *S. e.* = *Staphylococcus epidermidis*; RBC = red blood cells; and SN = supernatant. * Significant growth differences between the reference and the MAAC condition (Student *t*-test, α = 0.05, * *p* value < 0.05).

However, the antibacterial efficacy of MAAC applied at the surface of plasticized PVC in a nutrient broth medium may not be similar to that of plasticized PVC in LBP. The bacteria reduction factors were ≤90% in LBP (Table 2, Figure 4a and Figure S4A), which does not meet the ISO criteria for antibacterial efficacy [50].

These unexpected results were further investigated in additional experiments in which the MAAC’s antibacterial activity was assessed for each LBP component (i.e., RCC and PC). For the RCC, results suggest that part of the suboptimal antibacterial activity could be attributed to the high concentration of red blood cells. While *S. aureus* log reductions of ~0.7 were observed for the RCC resuspended in serum (ref = 5.3 ± 0.1 log _CFU/mL_, MAAC = 4.8 ± 0.2 log _CFU/mL_; *p* = 0.009) or saline (ref = 5.4 ± 0.1 log _CFU/mL_, MAAC = 4.7 ± 0.2 log _CFU/mL_; *p* = 0.002), a 1.1 ± 0.4 log reduction was observed for the isolated RCC supernatant (ref = 5.9 ± 0.1 log _CFU/mL,_ MAAC = 4.5 ± 0.3 log _CFU/mL_) (Figure 4b and Figure S4B). The suboptimal antibacterial activity observed in the presence of red blood cells is consistent with the hypothesis of Martinez-Camora et al. who postulated that silanol groups at the surface of SiO_2_ NP may interact with red blood cell phospholipids and cause hemolysis [62]. Reducing the concentration of red blood cells (1 × 10^−9^ dilution factor) before their exposure to the MAAC-treated PVC-DEHP increased the observed log reduction to 1.3 ± 0.7 (*p* = 0.025) (Figure 4b and Figure S4B). Given the low magnitude of this increase, further research is needed to confirm this hypothesis. Currently, hemolysis monitoring is not possible because all tests were performed on the PM sections.

For the PC, the high platelet concentration and the plasma constituents may explain the suboptimal effectiveness of the MAAC. Indeed, reduction factors of <90% were observed for each isolated component. Specifically, log reductions of 0.8 ± 0.8 (ref = 6.5 ± 0.1 log _CFU/mL_, MAAC = 5.7 ± 0.7 log _CFU/mL_) and 0.4 ± 0.4 (ref = 6.4 ± 0.2 log _CFU/mL_, MAAC = 6.1 ± 0.3 log _CFU/mL_) were observed for platelets resuspended in PBS and plasma, respectively. These reduction factors are modestly larger than the ones obtained using PC samples (0.2 ± 0.2 log [ref = 6.5 ± 0.2 log _CFU/mL_, MAAC = 6.3 ± 0.2 log _CFU/mL_]; Table 3, Figure 4c and Figure S4C). The reduction factor remained < 90% when using a 1 × 10^−6^ dilution in PBS (Table 3, Figure 4c and Figure S4C). In complex matrices, viscosity can limit molecular and bacterial diffusion and consequently, their interaction with the coating. In addition, the adsorption of large, hydrophobic plasma proteins onto the MAAC surface could hinder its antibacterial activity [63,64,65].

In the present study, *E. coli* exhibited a slower growth than other species in the PC stored under reference conditions (Figure 4a) which, as mentioned earlier, may reduce the calculated antibacterial activity (Equation (1)) [50]. Similar results were obtained using different strains of *E. coli* that are known to grow in PC, indicating that these results were not strain-specific (data not shown). These findings are consistent with *E. coli* presenting growth difficulties in PC. Platelets can internalize bacteria via TLR-4 receptors and release microbicidal proteins upon activation, leading to bacterial death [66].

The MAAC-induced, two-log reduction in bacterial load (observed in the NB) could be interesting for use in blood banking operations and medical applications [19]. Indeed, the contamination occurring during blood collection is usually not greater than 100 CFU [19]. Consequently, a two-log reduction could be of interest in reducing the risk of TTI [19]. However, experiments were performed using small volumes of LBP over a 24 h exposure, in line with ISO standards. These conditions may not adequately represent routine blood bank operations. Further research is warranted to explore the effectiveness of the MAAC over longer exposures that better capture each product’s shelf life (i.e., 7 days for PC; 42 days for RCC). In addition, it would be interesting to evaluate the effectiveness of the MAAC applied to PVC sheets before the LBP storage bag manufacturing process.

### 3.3. MAAC In Vitro Cytotoxicity

Regardless of the cytotoxicity assay used, the viability of L929 cells was consistently ≥90% in the MAAC-treated samples (Table 4). This coating thus meets the ISO 10993-5 guidelines criterion of ≥70% cell viability [51]. Viability was ≥90% after 24 h in contact with the coating, as measured by the MTT colorimetry test. Moreover, the microscopy-based cell count test and the cytometry test both confirmed that the MAAC does not significantly affect cell growth. The presence of the non-adhesive MAAC, however, decreases cell adhesion, which is characterized by rounder cells under optical microscopy (Figure S5).

The low MAAC cytotoxicity suggests that the hydrophobic functionalized SiO_2_ NPs of the coating could specifically interact with the saccharides of the peptidoglycan and thus denature the bacteria membrane. This could explain the apparent superior MAAC performances against Gram-positive strains compared to Gram-negative strains. Conversely, as described by Capeletti et al., eukaryotic cells may be protected by the glycocalyx layer on their surface [61]. Further analyses of blood products would be needed to consider using the MAAC for blood banking or biomedical applications. Additional experiments are being conducted to assess the impact of the MAAC on RCC and PC quality markers. Tests are also underway to optimize the coating application on the inner walls of regular blood product storage bags. The MAAC coating by direct injection in blood bags may change the final chemical nature of the inner walls due to poor drying, in which case it may be necessary to develop a new manufacturing concept better suited to the MAAC.

## 4. Conclusions

According to the ISO 22196 safety and effectiveness criteria, the SiO_2_ nanoparticular MAAC is effective against a broad spectrum of potentially pathogenic Gram-positive and Gram-negative bacteria. In all cases, the MAAC antibacterial reduction was over 90% in nutrient broth. Despite these promising results and since the test conditions of the ISO 22196 do not allow a good representation of the actual conditions of use, additional experiments using real storage conditions are needed. Higher contact times and test volumes could help more thoroughly assess the MAAC’s potential for biomedical applications. Moreover, it would be relevant to document the antimicrobial spectrum of the MAAC for other contaminants, such as yeasts, molds and viruses.

Knowing that the antibacterial action of the MAAC is more pronounced when applied to polyurethane and silicone, its use could be optimal for application at the surface of different medical devices, for example, with urinary catheters to prevent cases of nosocomial infections.

## Figures and Tables

**Table 1:** Bacterial culture conditions for antibacterial activity testing.

Material		PVC-DEHP		PVC-BTHC		Polyurethane/Silicone
Incubation temperatures (°C) ^a^		35	4	35	22	35
Gram	Positive	*S. aureus*		*S. aureus*		*S. aureus*
		N/A		*S. epidermidis*		*E. faecalis*
	Negative	*E. coli*		*E. coli*		*E. coli*
		*S. marcescens*		N/A		*K. pneumoniae*

^a^ Incubation at ≥90% relative humidity for 24 h.

**Table 2:** Bacterial reductions of MAAC-treated polymeric materials.

Material	Bacteria	Medium	Log Reduction ^a^	Reduction (%) *
PVC-DEHP	*S. aureus*	NB	3.8 ± 0.6	99.99
		RCC	0.3 ± 0.6	<90
	*E. coli*	NB	3.1 ± 0.1	99.9
		RCC	1.6 ± 0.3	99
	*S. marcescens*	NB	3.9 ± 1.7	99.99
		RCC	1.6 ± 0.1	99
PVC-BTHC	*S. aureus*	NB	2.2 ± 2.1	99
		PC	0.0 ± 0.0	<90
	*E. coli*	NB	1.4 ± 2.1	90
		PC	0.2 ± 0.2	<90
	*S. epidermidis*	NB	1.4 ± 1.3	90
		PC	2.2 ± 0.2	99
PVC	*S. aureus*	NB	2.3 ± 1.3	99
	*E. coli*	NB	4.6 ± 1.5	99.999
Polyurethane	*S. aureus*	NB	4.9 ± 0.4	99.999
	*E. coli*	NB	4.0 ± 0.5	99.99
	*K. pneumoniae*	NB	4.6 ± 0.6	99.999
	*E. faecalis*	NB	2.9 ± 0.8	99.9
Silicone	*S. aureus*	NB	5.2 ± 0.8	99.999
	*E. coli*	NB	5.2 ± 1.9	99.999
	*K. pneumoniae*	NB	5.5 ± 1.1	99.9999
	*E. faecalis*	NB	3.6 ± 0.9	99.99

NB = nutrient broth; PC = platelet concentrate; RCC = red cell concentrate. ^a^ The first decimal of the mean log-reduction values and their associated standard deviations are shown for comparison purposes. * <90% = <1 log reduction; 90% = 1 log reduction; 99% = 2 log reduction; 99.9% = 3 log reduction; 99.99% = 4 log reduction; 99.999% = 5 log reduction; 99.9999% = 6 log reduction.

**Table 3:** *S. aureus* reductions of MAAC-treated plasticized PVC in blood components.

Medium	Dilution	Log reduction	Reduction (%) *
PC ^¥^	ND	0.2 ± 0.2	<90
Platelet ^¥^	ND	0.8 ± 0.8	90
	1 × 10^−3^	0.2 ± 0.2	<90
	1 × 10^−6^	0.2 ± 0.2	<90
Plasma ^¥^	ND	0.4 ± 0.4	<90
	1 × 10^−3^	0.2 ± 0.2	<90
	1 × 10^−6^	0.3 ± 0.2	<90
RCC ^€^	ND	0.7 ± 0.2	90
RBC ^€^	ND	0.7 ± 0.1	90
	1 × 10^−3^	1.2 ± 0.3	90
	1 × 10^−6^	1.0 ± 0.6	90
	1 × 10^−9^	1.3 ± 0.7	90
SN ^€^	ND	1.1 ± 0.4	90

PC = platelet concentrate; RCC = red cells concentrate; RBC = red blood cells; SN = supernatant; ND = no dilution; **^¥^** Tests associated with MAAC treated PVC-BTHC; ^€^ Tests associated with MAAC-treated PVC-DEHP; * <90% = <1 log reduction; 90% = 1 log reduction.

**Table 4:** Cytotoxicity of MAAC in L929 cells.

	Viability (%)		
	Colorimetry (MTT)	Microscopy (AO/DAPI)	Cytometry (7-AAD)
L929 control	N/A	94.1 ± 1.8	92.1 ± 2.8
L929 with MAAC	96.2 ± 6.6	91.5 ± 6.2	90.9 ± 2.6

## Data Availability

Raw data were generated at Hema-Quebec. Derived data supporting the findings of this study are available from the corresponding author D.B. upon request.

## Supplementary Materials

The following are available online at https://www.mdpi.com/article/10.3390/antibiotics11010107/s1, Figure S1. SEM images of PVC-BTHC (a), polyurethane (b), silicone (c) and PVC (d) untreated and MAAC treated samples. A smooth and uniform layer for MAAC coated PM can be observed at the surface. A delamination (images (a) and (c)) can be observed for MAAC treated PM. Figure S2. Bacterial reduction in nutrient broth (1/500) with an initial inoculum of 6 × 10^5^ CFU/mL after 24 hours in contact with untreated PM (reference) and MAAC treated PM (*n* = 3). *S. a.* = *Staphylococcus aureus; E. c.* = *Escherichia coli; S. m.*= *Serratia marcescens; S. e.* = *Staphylococcus epidermidis; K. p.* = *Klebsiella pneumoniae; E. f.* = *Enterococcus faecalis*. Figure S3. Visualization of the antibacterial activity of MAAC on PVC-DEHP (a) untreated and (b) MAAC treated against a 6 × 10^5^ CFU/mL load of *S. aureus*. For the treated sample, 71 colonies were counted on the 10^0^ plate and 19 colonies on the 10^−2^ plate for the untreated sample. Smaller colonies are visible in the MAAC treated condition (b) compared to the untreated one (a). Figure S4. Bacterial reduction (mean ± standard deviation) in blood products with an initial inoculum of 6 × 10^5^ CFU/mL after 24 hours in contact with MAAC treated PM. (a) Bacterial reduction in RCC (PVC-DEHP) or PC (PVC-BTHC) matrices for three bacteria. Bacterial reduction of *S. aureus* in (b) RCC and (c) PC component, dilutions in saline (*n* = 3). *S. a* = *Staphylococcus aureus; E. c.* = *Escherichia coli; S. m.*= *Serratia marcescens; S. e.* = *Staphylococcus epidermidis*; RBC = red blood cells; SN = supernatant. Figure S5. L929 cell growth following 24 hours in presence or absence of MAAC. The initial inoculum was 1 × 10^5^ cells/well. Final volume was 2 mL per well. The incubation period was fixed at 24 h at 37 °C, 5% CO_2_. (a) Control condition (in contact with the bottom of the well), (b) test condition (in contact with a glass coverslip treated with 0.1 mL of liquid MAAC) and (c) negative control (in contact with a glass coverslip coated with 0.1 mL of liquid MAAC). All images were taken at a 100 X magnification. Table S1. Diameter of silica NP by DLS. Table S2. Dried thickness of the MAAC applied on textured PVC-DEHP by SEM. Table S3. Atomic distribution at the surface of MAAC treated and untreated PVC-DEHP by EDS.
