# Supplementary material for: Characterization of the Antibacterial Activity of an SiO2 Nanoparticular Coating to Prevent Bacterial Contamination in Blood Products
Source: Antibiotics (Basel). 2022 Jan 14;11(1):107. doi: 10.3390/antibiotics11010107 (PMC8773057; doi:10.3390/antibiotics11010107)
Supplement: Supplementary file 1 [file antibiotics-11-00107-s001.zip › antibiotics-1539828-supplementary.pdf]

Supporting information

Characterization of the Antibacterial Activity of an SiO_2 Nanoparticular Coating to Prevent Bacterial Contamination in Blood Products

Sahra Fonseca [1,2], Marie-Pierre Cayer [1], K.M. Tanvir Ahmmed [3], Nima Khadem-Mohtaram [3], Steve J. Charette [2] and Danny Brouard [1,4,*]

[1] Héma-Québec, Medical Affairs and Innovation, 1070, avenue des Sciences-de-la-Vie, Québec, QC G1V 5C3, Canada; Sahra.Fonseca@hema-quebec.qc.ca (S.F.); Marie-Pierre.Cayer@hema-quebec.qc.ca (M.-P.C.)

[2] Department of Biochemistry, Microbiology and Bioinformatics, Faculty of Science and Engineering, Laval University, Québec, QC G1V 0A6, Canada; Steve.Charette@bcm.ulaval.ca

[3] TriPhyll Inc., Mississauga, ON L4G 4X1, Canada; ahmmed.kmtanvir@gmail.com (K.M.T.A.); nkhadem@gmail.com (N.K.-M.)

[4] Department of Chemistry, Faculty of Science and Engineering, Laval University, Québec, QC G1V 0A6, Canada

* Correspondence: danny.brouard@hema-quebec.qc.ca

1. Materials and methods

1.1 Characterization techniques

Several characterization techniques were used to measure the abrasion and weatherability of the coating. The abrasion resistance was measured by the Taber abraser using ASTM D4060 test standard. Weatherability tests were conducted according to ASTM D 1654 and ASTM B 117 with no scribing for 100 hours to measure the salt spray resistance. UV resistance was measured according to ASTM D3424.

2. Results

2.2 Characterization

The abrasion resistance was calculated as loss in weight in mg at a specified number of abrasion cycles, according to ASTM D4060 standard. A rotary rubbing action was provided by an abrasive wheel at a specified load. A CS-17 wheel was used to produce abrasion. The CS-17 wheel was chosen as it produces a harsh abrasion. It has aluminum oxide or silicon carbide abrasive particles. A weight of 1000 g was applied on the wheel, and the reported weight loss was for 1000 cycle of run. For comparison, ASTM D4060 reported a 49.5 mg weight loss for polyurethane coating. The abrasion testing measured a weight loss of 27 mg after 1000 cycles of run. This shows that the coating is immune to regular wear and tear. In terms of weatherability, after 100 hours of salt spray testing, the coating received a rating of 10 out of 10, which shows that the coating is corrosion resistant in a controlled salt and humid environment. For the UV weatherability test, the coating had less than 1.3% gloss (60°) loss after 500 hours of UV radiation. This shows that the coating can resist UV and can deteriorate very slowly with prolonged exposure.

Table S1. Diameter of silica NP by DLS.

Spectra	Size (nm)	Intensity (%)	Standard Deviation (nm)
1	35.86	100	2.51
2	37.37	100	2.93

Front section area | Cross section area

(**a**) **Untreated**

MAAC treated

(**b**) **Untreated**

MAAC treated

(**c**) **Untreated**

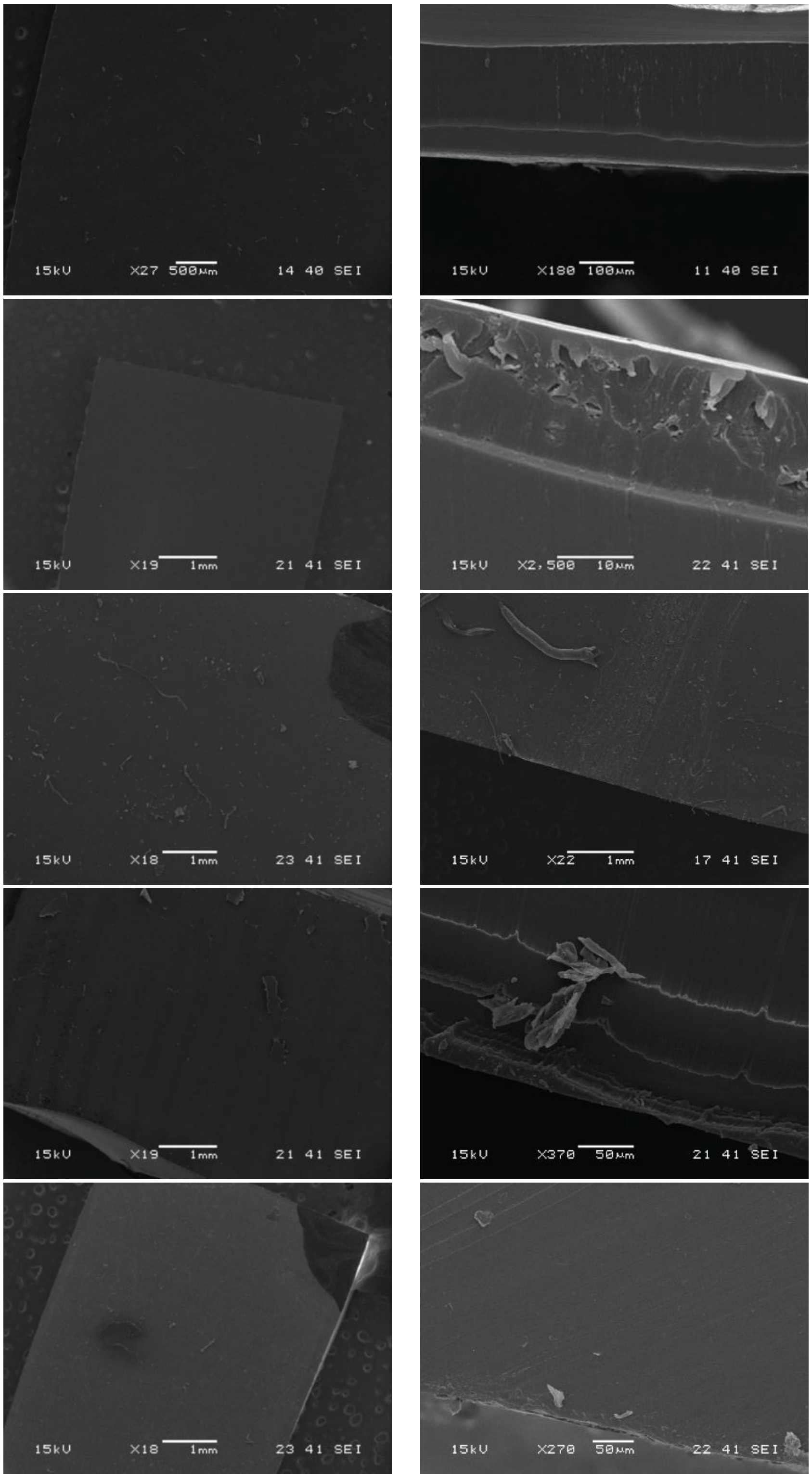

MAAC treated

(**d**) **Untreated**

MAAC treated

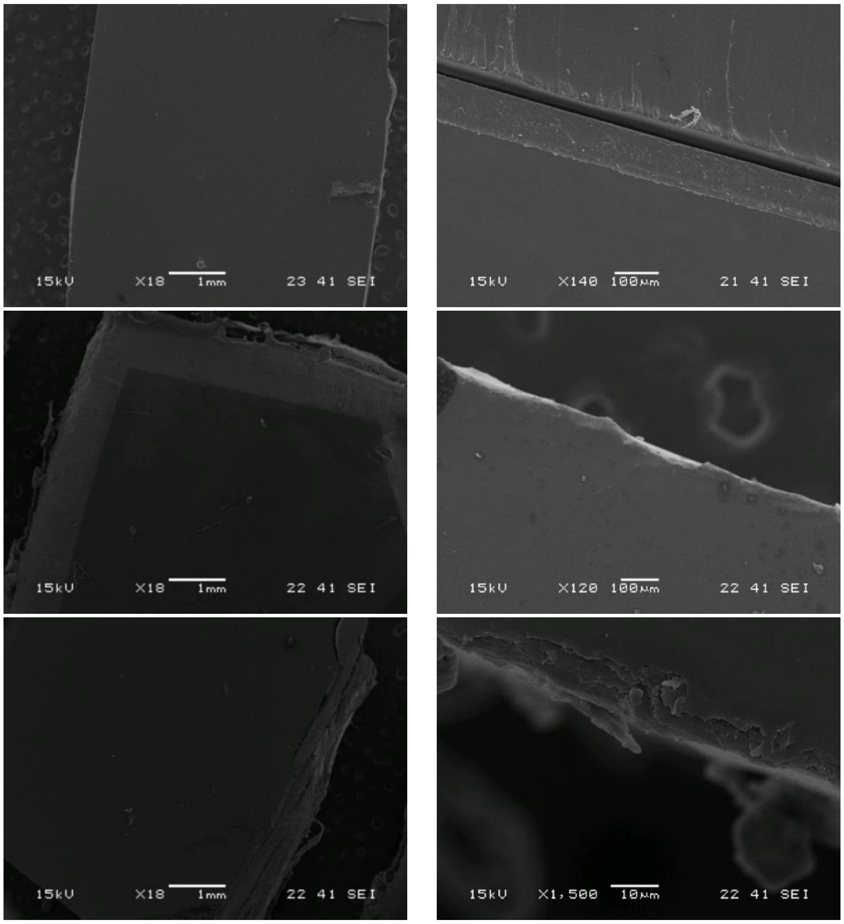

Figure S1. SEM images of PVC-BTHC (**a**), polyurethane (**b**), silicone (**c**) and PVC (**d**) untreated and MAAC treated samples. A smooth and uniform layer for MAAC coated PM can be observed at the surface. A delamination (images (**a**) and (**c**)) can be observed for MAAC treated PM.

Table S2. Dried thickness of the MAAC applied on textured PVC-DEHP by SEM.

PVC-DEHP section	MAAC thickness (µm)	Standard Deviation (µm)
Bottom	52	13
Upper	9	3

2.2 Antibacterial activity

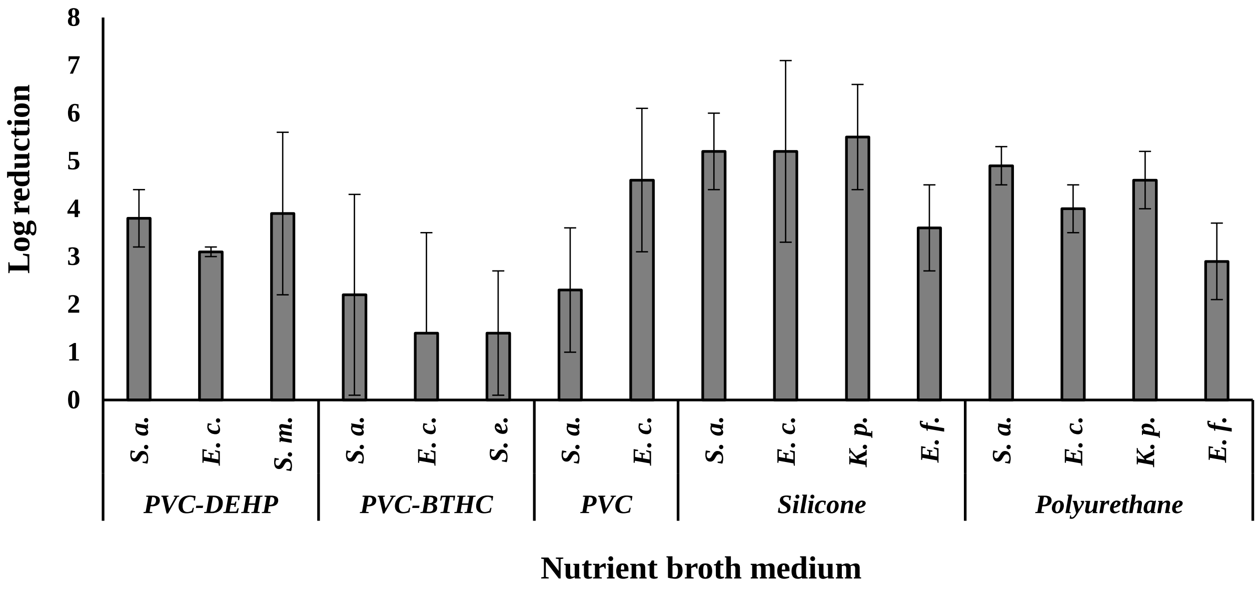

Figure S2. Bacterial reduction in nutrient broth (1/500) with an initial inoculum of 6 × 10^5 CFU/mL after 24 hours in contact with untreated PM (reference) and MAAC treated PM (n = 3*). S. a. = Staphylococcus aureus; E. c. = Escherichia coli; S. m.= Serratia marcescens; S. e. = Staphylococcus epidermidis; K. p. = Klebsiella pneumoniae; E. f. = Enterococcus faecalis.*

10^{0} 10^{-1} 10^{-2}

(**a**)

(**b**)

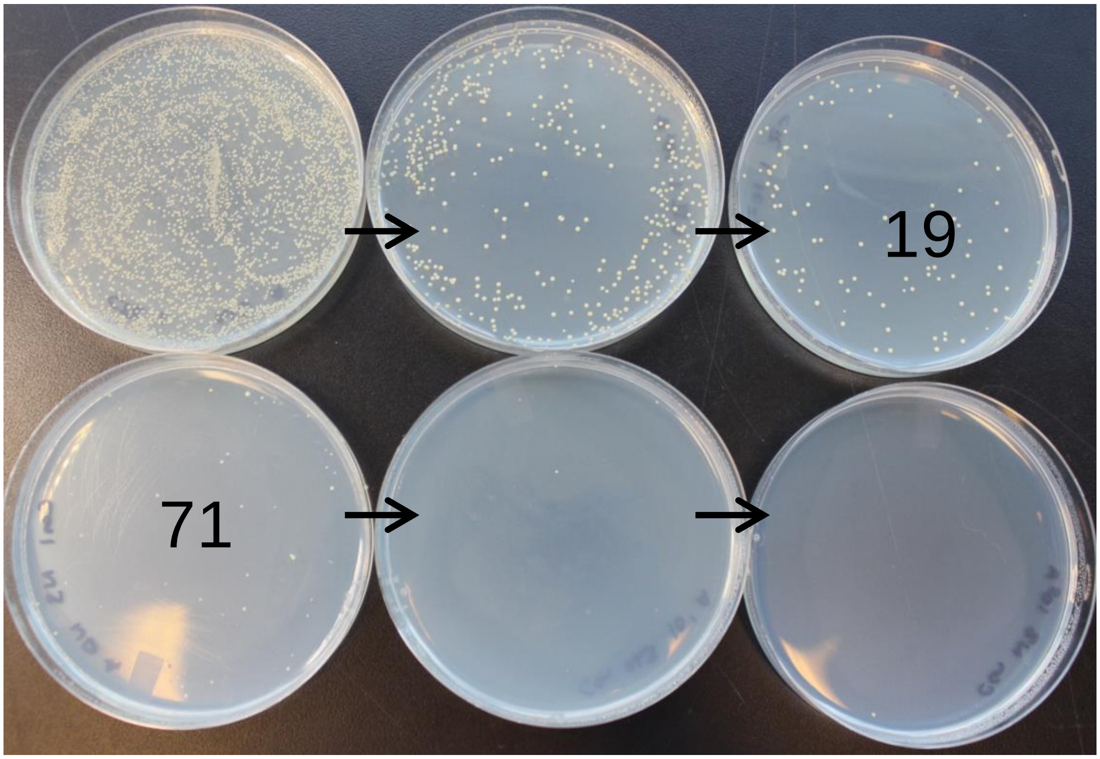

Figure S3. Visualization of the antibacterial activity of MAAC on PVC-DEHP (**a**) untreated and (**b**) MAAC treated against a 6 × 10^{5} CFU/mL load of *S. aureus*. For the treated sample, 71 colonies were counted on the 10^{0} plate and 19 colonies on the 10^{-2} plate for the untreated sample. Smaller colonies are visible in the MAAC treated condition (**b**) compared to the untreated one (**a**).

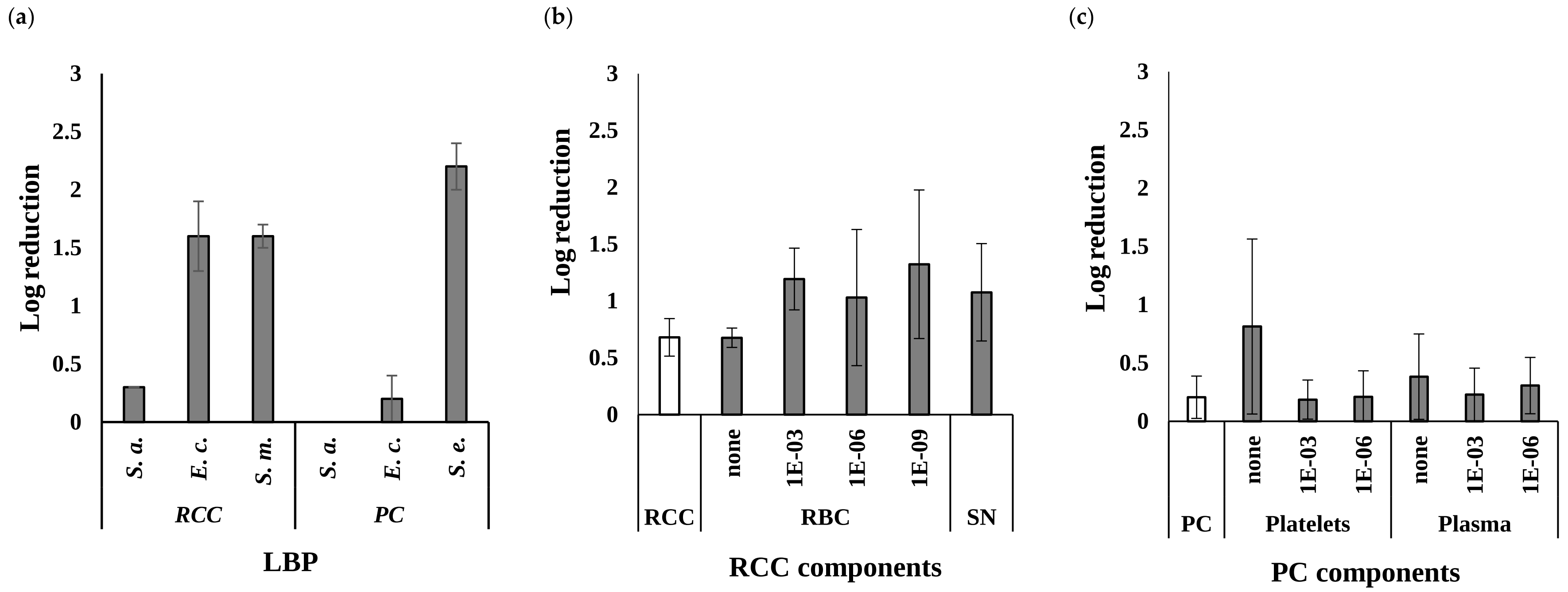

Figure S4. Bacterial reduction (mean ± standard deviation) in blood products with an initial inoculum of 6 × 10^5 CFU/mL after 24 hours in contact with MAAC treated PM. (**a**) Bacterial reduction in RCC (PVC-DEHP) or PC (PVC-BTHC) matrices for three bacteria. Bacterial reduction of *S. aureus* in (**b**) RCC and (**c**) PC component, dilutions in saline (n = 3). *S. a = Staphylococcus aureus; E. c. = Escherichia coli; S. m.= Serratia marcescens; S. e. = Staphylococcus epidermidis*; RBC = red blood cells; SN = supernatant.

2.3 MAAC in vitro cytotoxicity

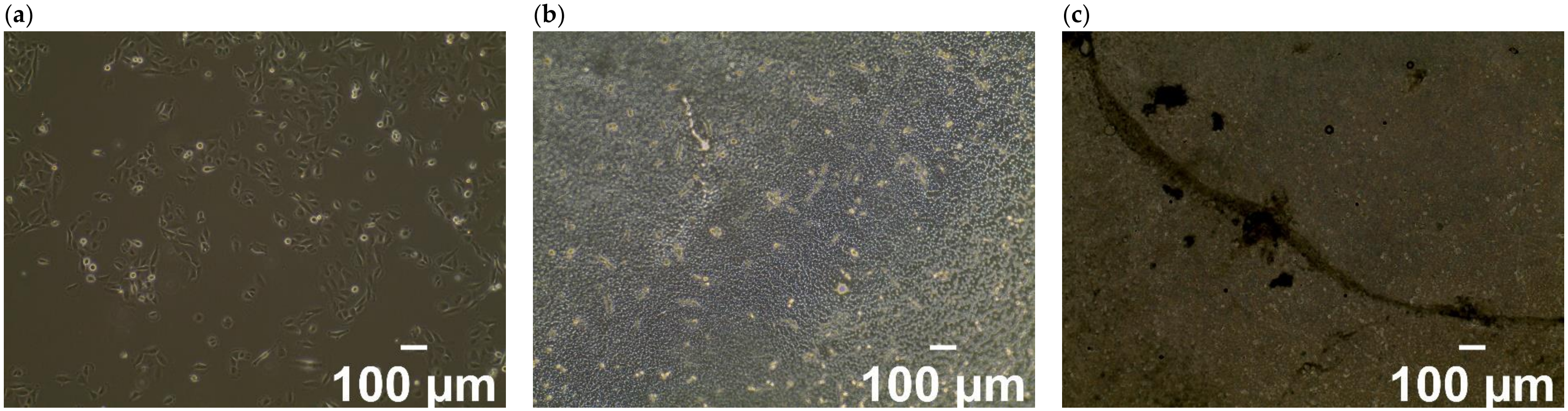

Figure S5. L929 cell growth following 24 hours in presence or absence of MAAC. The initial inoculum was 1 × 10^5 cells/well. Final volume was 2 mL per well. The incubation period was fixed at 24 h at 37 °C, 5% CO_2. (**a**) Control condition (in contact with the bottom of the well), (**b**) test condition (in contact with a glass coverslip treated with 0.1 mL of liquid MAAC) and (**c**) negative control (in contact with a glass coverslip coated with 0.1mL of liquid MAAC). All images were taken at a 100 X magnification.

Table S3. Atomic distribution at the surface of MAAC treated and untreated PVC-DEHP by EDS.

**material**	**% Atomic***			
	**C**	**O**	**Cl**	**Si**
PVC-DEHP	85 ± 2	8 ± 2	8 ± 1	0 ± 0
PVC-DEHP + MAAC	87 ± 3	12 ± 3	0 ± 0	2 ± 1

* Mean ± standard deviation.
